# Enhanced Electron Uptake and Methane Production by Corrosive Methanogens during Electromethanogenesis

**DOI:** 10.3390/microorganisms10112237

**Published:** 2022-11-12

**Authors:** Florian Mayer, Björn Sabel-Becker, Dirk Holtmann

**Affiliations:** 1 DECHEMA-Forschungsinstitut, Industrielle Biotechnologie, 60486 Frankfurt am Main, Germany; 2 Royal Netherlands Institute for Sea Research, Department of Marine Microbiology & Biogeochemistry, 1797 SZ Den Hoorn, Texel, The Netherlands; 3 Technische Hochschule Mittelhessen, Institut für Bioverfahrenstechnik und Pharmazeutische Technologie, 35390 Gießen, Germany

**Keywords:** electromethanogenesis, microbial electrosynthesis, corrosive methanogens, electron uptake mechanism, genome analysis, biofuel

## Abstract

Electromethanogenesis is an interesting next-generation technology to produce methane from CO_2_ and electricity by using methanogens. Iron-corroding methanogens might be of special interest for that application due to their natural ability for electron uptake. *Methanococcus maripaludis* Mic1c10 and KA1 were tested in bioelectrochemical systems. Strain Mic1c10 showed a 120% higher current density and an 84% higher methane production rate (16.2 mmol m^−2^ d^−2^) than the non-corrosive strain *Methanococcus maripaludis* S2, which was identified earlier as the best methane producer under the same experimental conditions. Interestingly, strain KA1 also showed a 265% higher current density than strain S2. Deposits at the cathodes were detected and analyzed, which were not described earlier. A comparative genome analysis between the corrosive methanogen and the S2 strain enables new insights into proteins that are involved in enhanced electron transfer.

## 1. Introduction

Due to climate change and a limited supply of fossil energy forms, other promising and sustainable sources, like renewables, are being more and more favored. Bio-based methane might be a potential biofuel to replace fossil energy sources. It can be used for transportation or heating and methane is also an important commodity for industrial applications today [1]. On the other hand, methane is also a climate gas that causes climate change and produces CO_2_ if burned as a fuel. Therefore, processes in which methane is produced from CO_2_, are of special interest to gain a zero-carbon reaction. Today, biological methane production is based mostly on biomass (e.g., wastes, crops), which is used in biogas plants. Thereby, biomass is converted anaerobically during hydrolysis, acidogenesis, acetogenesis, and methanogenesis to biogas. The obtained biogas consists of 50–60% methane, 40–50% CO_2,_ and impurities, depending on the used substrates [2,3]. A promising technology for the biological synthesis of methane, which does not produce additional CO_2_, is electromethanogenesis [4]. In this application, which relies on the microbial electrosynthesis (MES) process, methanogens take up electrons from a cathode and convert CO_2_ to methane. In times of growing interest in renewable energies, MES has the capability to store surplus current (or electrical energy) from different power sources (e.g., wind, water, solar) into chemicals [3,5,6]. In MES, at least two electrodes, a cathode and an anode, are used in a bioreactor, e.g., in an H-cell. The two chambers can be separated with a semi-permeable membrane that allows ion (e.g., proton) diffusion. At the cathode, a negative potential is applied. Due to water-splitting at the anode, protons can diffuse through the membrane into the cathode chamber, which are used by microorganisms, together with electrons, to reduce CO_2_ [5,7]. Depending on the microbial biocatalyst in the cathode chamber, several products from CO_2_ can be synthesized. One of these products is methane [4,8]. Mixed cultures with different bacteria and archaea, as well as a few methanogenic wild-type strains, were investigated for their ability to produce methane by MES [9]. In a mixed culture used for MES of methane, the methanogen *Methanobacterium palustre* could be identified as the most abundant strain. It was postulated that *M. palustre* takes up electrons directly from the electrode by forming a biofilm at the cathode [4]. This type of electron uptake is known as direct electron transfer (DET). A DET mechanism was also suggested for the strain *Methanococcus maripaludis* S2 during MES, if its pre-culture has been cultivated on formate and H_2_/CO_2_. *M. maripaludis* S2 released enzymes during MES to the cathode and the extracellular enzymes synthesized H_2_ and formate, which could be used for methane production by methanogenesis afterward [10]. It was shown, that this strain produced only H_2_ and methane during MES if the pre-culture was not cultivated on formate, but on H_2_/CO_2_. In this investigation, no cells could be observed at the cathode during MES, as shown by scanning electron microscopy (SEM) [11]. Using *M. maripaludis* S2 as a model organism, the scale-up of an electromethanogenic process in a bubble column reactor in a 50 L scale was shown [12,13]. Additionally, several different hydrogenotrophic methanogenic species were investigated for their ability to accept electrons from a cathode and reduce CO_2_ to methane by MES [11]. It could be shown that quite different methanogenic strains, namely *Methanococcus vannielii*, *Methanobacterium congolense*, *Methanolacinia petrolearia,* and *Methanoculleus submarines*, isolated from different habitats and having different morphologies, were able to take up electrons from a cathode at a negative potential of −700 mV vs. standard hydrogen electrode (SHE) and to convert CO_2_ to methane by MES [11]. Also, *Methanosarcina barkeri*, which is performing acetoclastic methanogenesis (methane production from acetate), is able to synthesize methane by MES [14]. Consequently, it seems that the electroactivity is not only restricted to hydrogenotrophic methanogens. Previously, the iron-corroding *Methanobacterium*-like strain IM1 [15,16] showed current uptake at −400 mV vs. SHE followed by methanogenesis [17]. For methane production also defined co-cultures, consisting of the iron-corroding sulfate-reducing strain IS4 (‘Desulfopila corrodens’), which produces H_2_ from Fe(0) [15], and *M. maripaludis*, which produces methane from H_2_ and CO_2_ were investigated [18]. However, so far IM1 is the only iron-corroding methanogen that has been tested in pure culture for the MES of methane. Other methanogens have also shown corrosive characteristics, but have not been tested in MES [19]. The strains *Methanococcus maripaludis* Mic1c10 and KA1 are highly interesting as they have demonstrated corrosive activity compared to non-corrosive *M. maripaludis* strains (e.g., S2, C5, C6, and C7) [20,21,22]. The proposed mechanism of the corrosive property for both strains is based on an unstable 12 kb genomic island called “MIC-Island”. It encodes a secretion system and a unique [NiFe] hydrogenase, which is supposed to be on a redox-active surface enabling hydrogen production via metal oxidation [23,24]. Iron-corroding methanogens might be of special interest for MES since the iron corrosion mechanism is an electron uptake process in which the microorganism is leaching electrons out of a material, e.g., Fe^0^, and forms a corrosion end product on the material.

Therefore, the aim of this study was to investigate further iron-corroding methanogens, *Methanococcus maripaludis* Mic1c10 and KA1, for their ability to take up electrons from a cathode and to reduce CO_2_ to methane via MES. We compared the results with non-corrosive methanogens to identify strains with the highest methane production and current uptake rates.

## 2. Materials and Methods

### 2.1. Strains and Culture Conditions

*Methanococcus maripaludis* Mic1c10 and KA1 were obtained by the BAM Federal Institute for materials research and testing (Berlin, Germany). The strains were cultivated in 250 mL septum flasks with 50 mL or in 500 mL septum flasks (Glasgerätebau Ochs, Bovenden, Germany) with 200 mL M141 medium and H_2_/CO_2_ (80/20, *v*/*v*) as a gas phase, pressurized to 2.1 bar. Medium composition, preparation, and cultivation were described earlier in detail [11].

### 2.2. Reactor Setup and Culture Conditions for Electromethanogenesis

**Figure 1:**
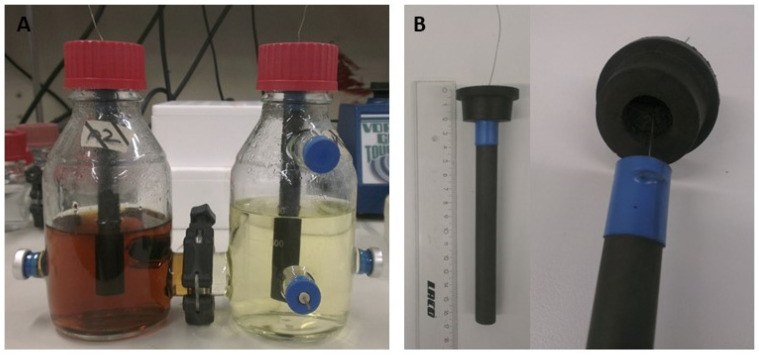
Bioelectrochemical setup with (**A**) custom-made, gas-tight 500 mL H-cell with Balch tube connections and (**B**) electrode integration via a platinum wire. Reprinted with permission from Ref. [11]. 2019, Elsevier.

For electromethanogenesis, custom-made gas-tight H-cells (Fischer Labortechnik, Frankfurt, Germany) with a maximum volume of 500 mL and with Balch tube connections for gas sampling and reference integration were used (Figure 1). Briefly, graphite rods (15 cm in length; 1.4 cm in diameter; 4.5 cm of electrode submerged into the media) were used in the gas-tight H-cells. The electrodes were connected with a platinum wire (guided through a gas-tight rubber stopper) to the potentiostat (IPS Elektroniklabor GmbH & Co. KG, Münster, Germany). Both electrodes were separated by a Nafion 117 proton exchange membrane (QuinTech, Göppingen, Germany). A platinum electrode (inner diameter 3 mm, outer diameter 6 mm, ALS Co., Ltd., Tokyo, Japan) was used as a reference and integrated via a Balch tube connection into the cathode chamber. Potentials mentioned in this publication were reported against standard hydrogen electrode (SHE). In this bioelectrochemical setup, experiments were performed in a minimal medium (MM141), exactly as previously described [11], and a potential of −700 mV vs. SHE was applied. Anode and cathode chambers were pressurized to 1.7 bar with a gas phase of N_2_/CO_2_ (80/20, *v*/*v*) to improve gas availability in the solution for the methanogens, as described earlier [25]. The inoculation density at the cathode was 0.1 (OD_600nm_). H-cells were operated at 37 °C using a heating hood and the solution was stirred at 100 rpm. During the chronoamperometric measurement (CAM), current over time was measured with 30 data points per hour. The Coulombic efficiency (CE) was calculated according to the following equation:

$$\mathrm{CE}=n_{\mathrm{GC}}/n_{\mathrm{CAM}}\;\mathrm{with}\;n_{\mathrm{CAM}}\;=\;\int_{t=0}^{t=end}I\;dt/F\;\times \;z$$

whereby n_GC_ is the amount of substance (methane or hydrogen) measured by gas chromatography, n_CAM_ is the maximum possible amount of substance (methane or hydrogen) calculated with the current density, I, the time, t, the Faraday constant, F, and the number of electrons transferred, z (8 electrons for methane, 2 electrons for hydrogen).

### 2.3. Gas Analytics

Gas analytics were performed with a 490 Micro-GC system (Agilent Technologies, Santa Clara, CA, USA) and gas samples were analyzed by injection into a capillary GC column system and a micro-machined thermal conductivity detector (µTCD), as described earlier [11].

### 2.4. Scanning Electron Microscopy (SEM)

For visualization of cells on the electrode surface, cells were fixed with glutaraldehyde and dehydrated with ethanol, as described earlier [26]. After dehydration, electrodes were dried on air for 24 h and afterward for 7 days in an exsiccator in the presence of Orange-Gel (Merck, Darmstadt, Germany). A FlexSEM 1000 II scanning electron microscope (Hitachi, Japan) was used for electrode surface visualization.

### 2.5. Element Measurements and Mapping by EDX

Element measurements were performed by energy-dispersive X-ray spectroscopy during scanning electron microscopy with a Z2 Analyzer system (EDAX Inc., Mahwah, NJ, USA). For element measurements at different points on the electrode, the element composition in three spots of two different areas was measured. The working distance between the object table and detector was set to 10 mm and the voltage was set to 15 keV. For element mapping, an area at the electrode was defined by its size, and then the element distribution was measured in this area by scanning 128 frames.

### 2.6. Genome Analysis of M. maripaludis Strains S2 and KA1

Genomes of the strains *M. maripaludis* S2 and KA1 were used from the database NCBI for analysis. The genome of non-corrosive *M. maripaludis* S2 (entry: NC_005791.1) was aligned against the genome of corrosive *M. maripaludis* KA1 (entry: NZ_AP011526.1) using the program Geneious 9.1.5 and the LASTZ sequence alignment program in version 1.02.00 (released 12 January 2010) [27,28].

## 3. Results and Discussion

### 3.1. Electromethanogenesis with M. maripaludis Strains Mic1c10

**Figure 2:**
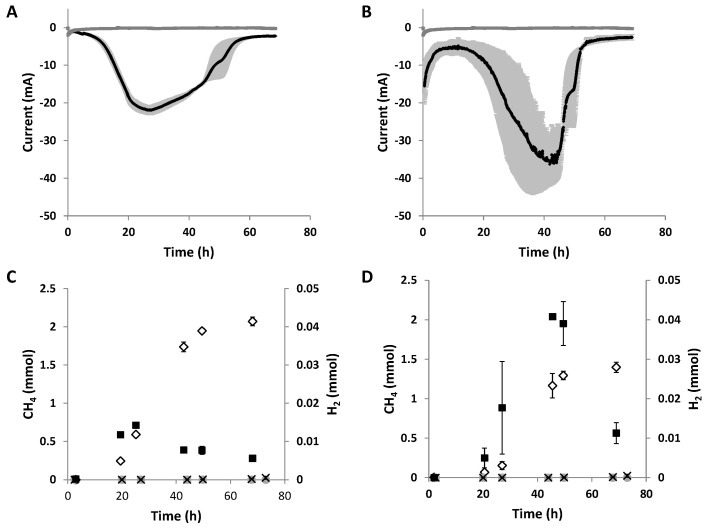
Current consumption during electromethanogenesis with (**A**) *M. maripaludis* Mic1c10 and (**B**) *M. maripaludis* KA1. The current consumption of the biological sample (black) and abiotic control (dark grey) is shown. The mean value of two replicates with mean deviation (light grey) is displayed. Methane (white diamonds) and H_2_ (black squares) production during electromethanogenesis in (**C**) *M. maripaludis* Mic1c10 and (**D**) *M. maripaludis* KA1. Methane and H_2_ production in abiotic controls of (**C**,**D**) are displayed with grey circles and black crosses, respectively. The mean value of two replicates with mean deviation is shown.

In order to further expand the spectrum of corrosive microorganisms for MES, two iron-corroding methanogens, *M. maripaludis* Mic1c10 [20] and *M. maripaludis* KA1 [21] were tested for CO_2_ reduction to methane with electrons provided at the cathode. *M. maripaludis* Mic1c10 took up electrons at a potential of −700 mV vs. SHE directly after the start of the experiment, as shown by the chronoamperometric measurement (Figure 2A). Methane was formed up to 2.07 (±0.06) mmol (Figure 2C), parallel to the current uptake. Identical to the different hydrogenotrophic strains *Methanococcus vannielii*, *Methanococcus maripaludis* S2, *Methanolacinia petrolearia*, *Methanobacterium congolense*, and *Methanoculleus submarinus* tested prior [11], hydrogen was also formed during electromethanogenesis. The maximal current density for *M. maripaludis* Mic1c10 during electromethanogenesis was 487.13 (±27.24) mA m^−2^ (geometrical surface area of the cathode) and more than 120% higher than for the *M. maripaludis* S2 strain tested in the same system (Table 1). The methane production rate was 16.2 (±0.45) mmol m^−2^ d^−2^ for strain Mic1c10 (Table 1) and therefore 84% higher than with the best methane producer *M. maripaludis* S2 identified earlier [11]. Although, the Coulombic efficiency for methane was almost similar, which was 61.3 (±1.5)% compared to *M. maripaludis* S2, which was 58.9 (±0.8)%. Hence, the higher current density enabled a higher production of methane using strain Mic1c10 but with a similar Coulombic efficiency compared to strain S2. Control experiments without cells showed as good as no current uptake, no methane production, and only a very low abiotic hydrogen production on graphite electrodes at −700 mV vs. SHE (Figure 2A,C). The abiotic hydrogen production was not enough to explain the amount of methane produced during electromethanogenesis. An indirect electron pathway (IET), which is based on the production of abiotic hydrogen and the subsequent use of this hydrogen in a second step by the microorganism as an electron source to reduce CO_2_ to methane [8,10], can be excluded. However, an IET based on secreted hydrogenases interacting with the electrode surface and producing small intermediates (e.g., H_2_, formate) might be one explanation for the high electron uptake [24]. Since only small amounts of hydrogen were measured, the consumption of intermediates had to take place directly after generation. As *M. maripaludis* belongs to the order *Methanococcales*, its H_2_ threshold is very low (H_2_ partial pressure < 10 Pa), which could explain the small amounts of H_2_ in the headspace of the reactor [29]. A direct electron pathway (DET) for *M. maripaludis* Mic1c10 in which the corrosive methanogen is taking up electrons from the cathode is another possibility. Here, biotic H_2_ is also produced from electrons and protons and CO_2_ is reduced to methane. Such a kind of mechanism has been proposed already for other hydrogenotrophic methanogens during electromethanogenesis [11].

### 3.2. Electromethanogenesis with M. maripaludis Strains KA1

The second iron-corroding methanogen tested in this study, *M. maripaludis* KA1, was isolated from a bottom plate of a raw-oil storage tank [21]. The strain KA1 took up electrons from a cathode at a negative potential of −700 mV vs. SHE (Figure 2B). Electron uptake during electromethanogenesis with *M. maripaludis* KA1 started after an adaptation/polarisation phase of 14 h. Simultaneously with current production, *M. maripaludis* KA1 produces methane up to 1.4 (±0.07) mmol (Figure 2D). Additionally, here biotic hydrogen is formed during electromethanogenesis. The maximal current density obtained during electromethanogenesis with *M. maripaludis* KA1 was 800.89 (±115.87) mA m^−2^ (Table 1). Again, this is a 64% higher current density than with strain Mic1c10 and a 265% higher current density than obtained with strain *M. maripaludis* S2. The methane production rate was 10.8 (±0.51) mmol m^−2^ d^−1^ and indeed was higher than for *M. maripaludis* S2, but around 30% lower than the methane production rate with strain Mic1c10 (Table 1). Therefore, the Coulombic efficiency is also lower, at 35.1 (±11.8)%. Explanations for this low efficiency can be of biological or electrochemical nature; it might be possible that electrons are used not only for electromethanogenesis, but also for other, as of yet unknown, side reactions in strain KA1. A possible electron sink might be formate. It was shown prior that formate was found in cultures of *M. maripaludis* S2 during MES when the used pre-culture was grown on H_2_/CO_2_ and formate [24]. Bioelectrochemical H_2_ formation is in general also possible, but the rapid consumption of hydrogen hampers the balancing of H_2_, which results in a Coulombic efficiency for H_2_ far below 1% (Table 1). Other possible electron sinks that might have caused the low CE for methane production could be acetyl-CoA synthesis as a central metabolite for cellular compounds, glycogen assimilation as a storage substance, and energy-intensive nitrogen fixation [30,31,32]. Possible electrochemical side reactions, responsible for the low Coulombic efficiency, might be also corrosion of the electrode at a potential of −700 mV vs. SHE or ion polarization in the medium. Again, the control experiments without cells showed no current uptake, no methane production, and only a very small abiotic hydrogen production at a potential of −700 mV vs. SHE.

### 3.3. Visualization of the Cathodes after Electromethanogenesis

**Figure 3:**
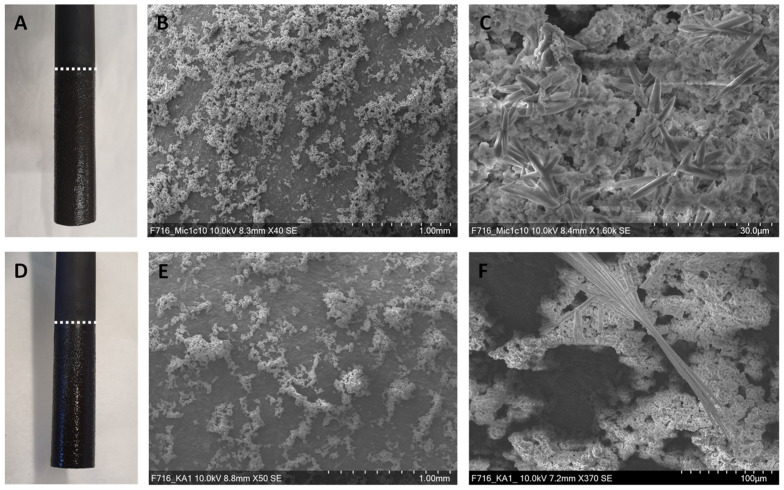
Photographs of the cathodes after electromethanogenesis with (**A**) *M. maripaludis* Mic1c10 and (**D**) *M. maripaludis* KA1. SEM pictures of cathodes used in electromethanogenesis with strain Mic1c10 (**B**,**C**) and strain KA1 (**E**,**F**).

In order to investigate cell attachment and biofilm formation, the cathodes used during electromethanogenesis with *M. maripaludis* Mic1c10 and strain KA1 were examined by scanning electron microscopy (SEM). Already visible to the naked eye was a deposit on the submerged part of the cathodes for both corrosive methanogens (Figure 3A,D). Unexpectedly, no cells, neither from strain Mic1c10 nor from strain KA1, could be detected on the cathodes by SEM (Figure 3B,C,E,F), although *M. maripaludis* is capable of attaching to surfaces [33]. Additionally, for the non-corrosive methanogens *M. vannielii*, *M. submarinus*, *M. petrolearia*, and *M. maripaludis* S2, no cell attachment or biofilm formation could be observed, as described earlier [11]. Cell attachment was only observed for M. congolense [34] on the similar graphite electrodes [11] used for the corrosive methanogens in this study. This finding strongly suggests that secreted soluble hydrogenases enabled higher electron uptake [24]. Surprisingly, the deposit on the cathodes of Mic1c10 and KA1 was of chemical nature. Different types of deposits, distributed over the whole submerged area of the cathodes, could be observed on the surface (Figure 3C,F). Such a deposit was not reported on the cathodes after electromethanogenesis for the non-corrosive methanogens [11]. Cathodes from control experiments with an applied potential of −700 mV vs. SHE, but without cells, showed no deposit formation on the surface of the electrode. Additionally, cathodes from H-cells in which the corrosive methanogenic strains Mic1c10 and K1 were cultivated without applying a negative potential showed no deposit formation on the surface of the electrode. These experiments demonstrate that a negative potential and corrosive methanogens are necessary to form the deposit on the electrode and is most likely caused by a local increase of the pH at the cathode-media-interface at negative potentials. As a result, compounds of the MM141 medium deposit on the cathode as the EDX analysis suggested.

### 3.4. Element Analysis of Deposits Formed during Electromethanogenesis

**Figure 4:**
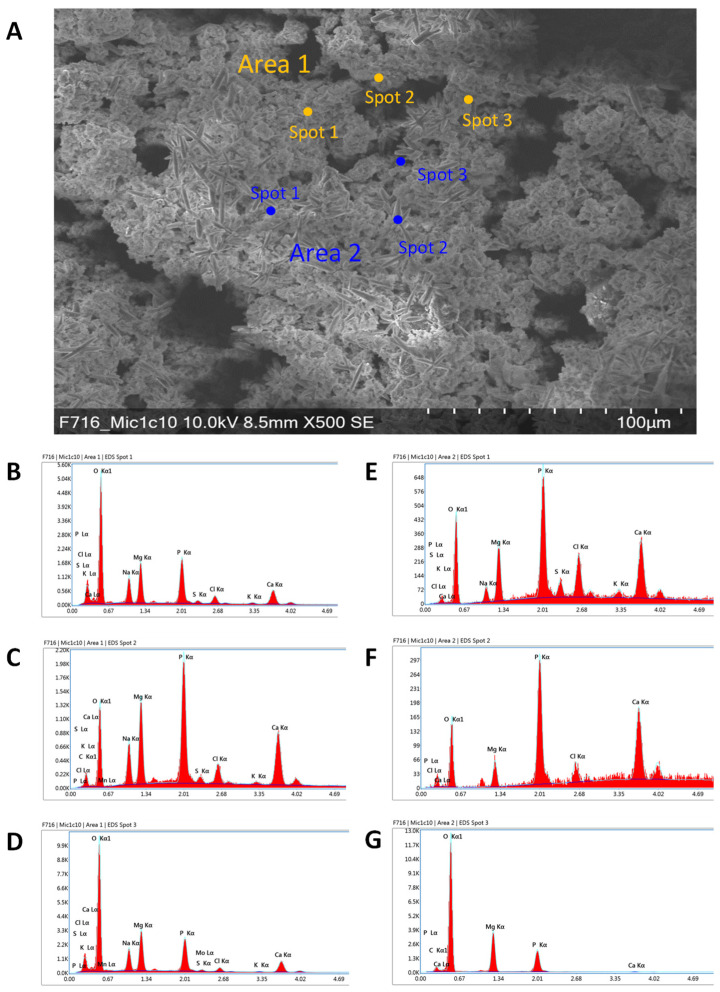
Element measurements at different spots of two areas of the electrode used during electromethanogenesis with *M. maripaludis* Mic1c10. (**A**) SEM picture of the electrode with deposits and distribution of the spots in areas 1 and 2. EDX analysis of (**B**) spot 1, (**C**) spot 2 and (**D**) spot 3 in area 1. EDX analysis of (**E**) spot 1, (**F**) spot 2 and (**G**) spot 3 of area 2. The x-axis shows the energy in keV and the y-axis shows the total counts.

In order to investigate the deposits on the surface of the cathodes from *M. maripaludis* Mic1c10 and KA1, the electrodes were analyzed by EDX during scanning electron microscopy. For *M. maripaludis* Mic1c10, mainly the elements oxygen (O), phosphor (P), sodium (Na), magnesium (Mg), chlorine (Cl), and calcium (Ca) were found in different amounts depending on the point of measurement on the electrode (Figure 4). For *M. maripaludis* KA1, the elements oxygen (O), phosphor (P), sodium (Na), magnesium (Mg), and calcium (Ca) were also found, and additionally, potassium (K) was identified (Figures S1 and S2). As of now, deposit formation on the cathode during electromethanogenesis has not been described before. Electromethanogenesis with the different hydrogenotrophic strains *M. vannielii*, *M. maripaludis* S2, *M. petrolearia*, *M. congolense*, and *M. submarinus* showed no deposit formation on the cathode [11]. Such a phenomenon was also not described by other groups who used the same minimal medium (MM141), e.g., during electromethanogenesis of *M. maripaludis* S2 [10,24] or during direct electron uptake of a purified heterodisulfide reductase supercomplex (Hdr-SC), which was adsorbed to an electrode and produced formate and H_2_ at −600 mV vs. SHE [35].

### 3.5. Compound Identification of Deposits by EDX Mapping

**Figure 5:**
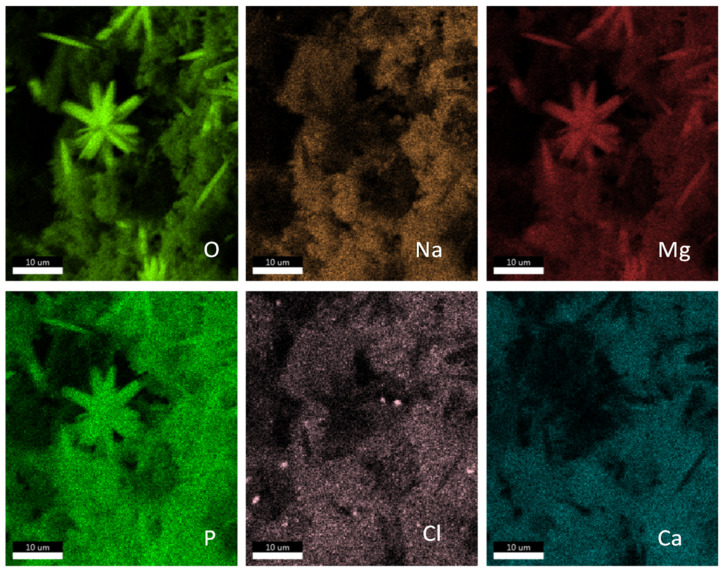
EDX mapping of an area with deposits of the electrode used during electromethanogenesis with *M. maripaludis* Mic1c10. Different elements are shown in different colors.

An EDX mapping with the cathodes from electromethanogenesis with *M. maripaludis* Mic1c10 and KA1 was performed to examine the distribution of the previously identified elements (3.4) and to identify possible chemical compounds. On the electrode from strain Mic1c10, some deposits consist of NaCl and CaCl, and others, e.g., the star-shaped deposit on the electrode, consist of magnesium phosphate (Figure 5). This was also confirmed by element analysis of that deposit on the electrode. During spot measurement, only the elements magnesium (Mg), oxygen (O), and phosphor (P) could be identified (Figure 4G). The deposits on the electrode from strain KA1 consist of magnesium phosphate too, but potassium and/or sodium phosphate might also be possible, as shown by EDX mapping (Figure S3). The chemical compounds identified in this study were not found before. During iron corrosion with the strain *M. maripaludis* KA1, FeCO_3_ could be identified as the end product on the surface of the iron coupons, in addition to a change of the iron surface [21]. A change in the surface of the graphite electrodes used during electromethanogenesis with *M. maripaludis* Mic1c10 and KA1 could not be observed with SEM. Nevertheless, corrosion of graphite electrodes is in general possible and has been shown previously [36,37], for example, with scanning electrochemical microscopy (SECM).

### 3.6. Comparison of the Genomes of M. maripaludis S2 and Strain KA1

We compared the genome of the non-corrosive S2 strain with the genome of the corrosive KA1 strain to hypothesize which genes (and their encoding proteins) might be responsible for the improved electron uptake of strain KA1. For *M. maripaludis* Mic1c10, no genomic data are available at the moment. Sequence alignment revealed that *M. maripaludis* KA1 has 10 additional genes encoding for iron-sulfur binding proteins in comparison to strain S2 (Table S1). Iron-sulfur clusters are known for their ability to transport electrons. For example, a few years ago it was shown that the formyl-methanofuran dehydrogenase, which catalyzes the first step of methanogenesis, contains 46 [4Fe-4S] clusters and functions as an electron relay for electron transport [38]. Besides iron-sulfur binding proteins, there is a higher content of ferredoxin-containing proteins (oxidoreductases) encoded in the genome of *M. maripaludis* KA1. Analysis of the genome comparison showed five additional genes in strain KA1. As it has been proposed for cytochrome C-free gram-positive bacteria, oxidoreductases, and membrane-bound iron-sulfur binding proteins are involved in electron uptake from a cathode [39]. Thus, it might be possible that these proteins, encoded by the additional genes in the genome of strain KA1, are responsible for its higher electron uptake rate. Above all, *M. maripaludis* does not contain cytochromes involved in electron transport and energy conservation [29,40]. In addition, two genes encoding for iron ABC transporter permeases were identified in the genome of KA1, which might also play a role in electron uptake, in addition to iron transport. These transmembrane proteins transport specific molecules, in the direction of a concentration gradient, into or out of the cell. In 2018, it was published that a DNA segment called “MIC-Island” is responsible for methane production during the cultivation of *M. maripaludis* OS7 on iron [22]. *M. maripaludis* S2 does not contain this DNA segment, but in the genome of KA1, the “MIC-Island” could be identified. As described for the strain OS7, the DNA segment consists of genes encoding for the twin-arginine translocation (TAT) pathway, a carbonic anhydrase, two [NiFe] hydrogenases, and a hydrogenase maturation protease [22]. These genes could also be found in the “MIC-Island” of strain KA1, but the genes for both of the [NiFe] hydrogenases are annotated as hypothetical proteins. Nevertheless, a BLAST search showed that these hypothetical proteins have a sequence identity of 27% and 30% to the [NiFe] hydrogenase of *M. vannielii* and to the F420-non-reducing hydrogenase of *M. maripaludis*, respectively. During the cultivation of *M. maripaludis* OS7 with iron particles, it has been postulated that the hydrogenases of the “MIC-Island” are translocated through the TAT pore to the particles and then produce H_2_, and afterward produce methane due to the reduction of the iron [22]. Despite the fact that the “MIC-Island” is also present in the genome of strain KA1, a similar mechanism is rather unlikely in *M. maripaludis* KA1 due to the fact that the electrode is occupied by different deposits.

## 4. Conclusions

The iron-corroding methanogens *M. maripaludis* Mic1c10 and KA1 showed a higher current density and a higher methane production rate during electromethanogenesis than the non-corrosive methanogens, e.g., *M. maripaludis* S2. Thereby, biotic H_2_ was also formed during MES, which seems to be an intermediate in the microbial electrosynthesis of methane. The amount of abiotic H_2_ was not enough to explain methane production only via an indirect electron uptake. No cells were attached to the cathodes and higher current densities in MES with corrosive *M. maripaludis* strains that carry the “MIC-Island” trait were observed. Additionally, deposits of different chemical compounds were formed at the electrode during electromethanogenesis, which have to be investigated further in the future. Iron-sulfur binding proteins, as well as ferredoxin-containing proteins, might be responsible for the enhanced electron uptake and methane production during electromethanogenesis with corrosive methanogens, as shown by our genome comparison. In general, the applied genome comparison shows that bioinformatics can be used to elucidate the performance of different organisms in MES and also make it possible to identify new electroactive organisms.

## Figures and Tables

**Table 1:** Methane and hydrogen production during MES with different *Methanococcus maripaludis* strains.

*Methanococcus* *maripaludis*	CH_4_ (mmol m^−2^ d^−1^) ^a^	H_2_ (mmol m^−2^ d^−1^) ^a^	Max. Current Density (mA m^−2^) ^a^	Coulombic Efficiency (%) for H_2_^a,b^	Coulombic Efficiency (%) for CH_4_^a,b^
Strain S2 ^c^	8.81 (±0.51)	0.06 (±0.01)	219.61 (±21.89)	0.10 (±0.03)	58.9 (±0.8)
Strain Mic1c10	16.20 (±0.45)	0.04 (±0.00)	487.13 (±27.24)	0.04 (±0.00)	61.3 (±1.5)
Strain KA1	10.80 (±0.51)	0.09 (±0.02)	800.89 (±115.87)	0.08 (±0.04)	35.1 (±11.8)

^a^ Each value is the mean value of two replicates with mean deviation as an error; ^b^ Coulombic efficiency was determined at the end of the experiment; ^c^ Data from [11].

## Data Availability

Not applicable.

## Supplementary Materials

The following supporting information can be downloaded at: https://www.mdpi.com/article/10.3390/microorganisms10112237/s1, Figure S1: Element measurements at different spots of the electrode used during electromethanogenesis with *M. maripaludis* KA1, Figure S2: Element measurements at different spots of the electrode used during electromethanogenesis with *M. maripaludis* KA1, Figure S3: EDX mapping of an area with deposits of the electrode used during electromethanogenesis with *M. maripaludis* KA1. Different elements are shown in different colors, in Table S1. Additional genes in the genome of *M. maripaludis* KA1 in comparison to strain S2.
