# Supplementary material for: Enhanced Electron Uptake and Methane Production by Corrosive Methanogens during Electromethanogenesis
Source: Microorganisms. 2022 Nov 12;10(11):2237. doi: 10.3390/microorganisms10112237 (PMC9698937; doi:10.3390/microorganisms10112237)
Supplement: Supplementary file 1 [file microorganisms-10-02237-s001.zip › microorganisms-1955703-supplementary.pdf]

Supplementary Materials

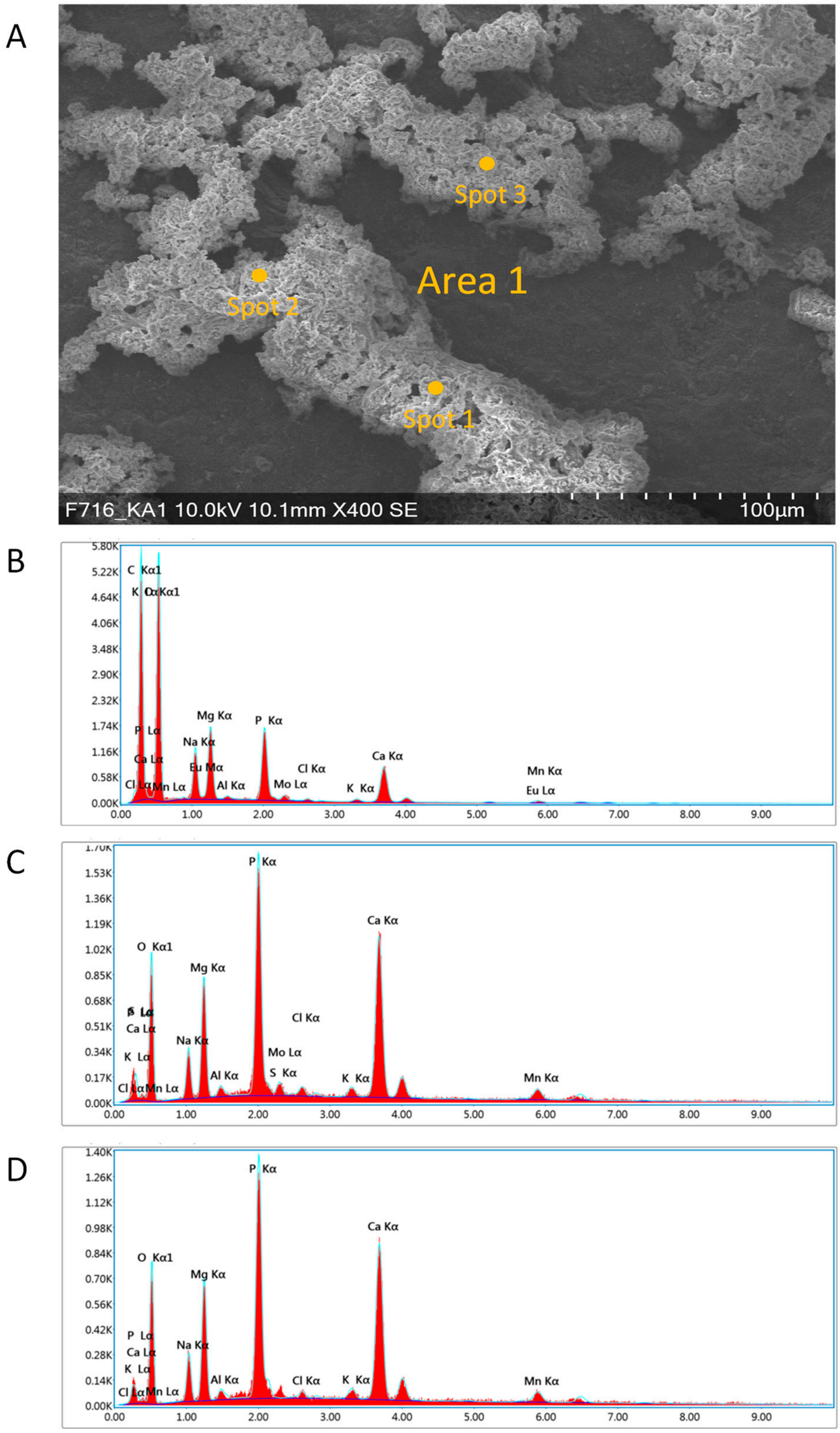

Figure S1. Element measurements at different spots of the electrode used during electromethanogenesis with *M. maripaludis* KA1. **(A)** SEM picture of electrode with deposits and distribution of the spots in area 1. **(B)** EDX analysis of spot 1, **(C)** EDX analysis of spot 2 and **(D)** EDX analysis of spot 3. The x-axis shows the energy in keV and the y-axis shows the total counts.

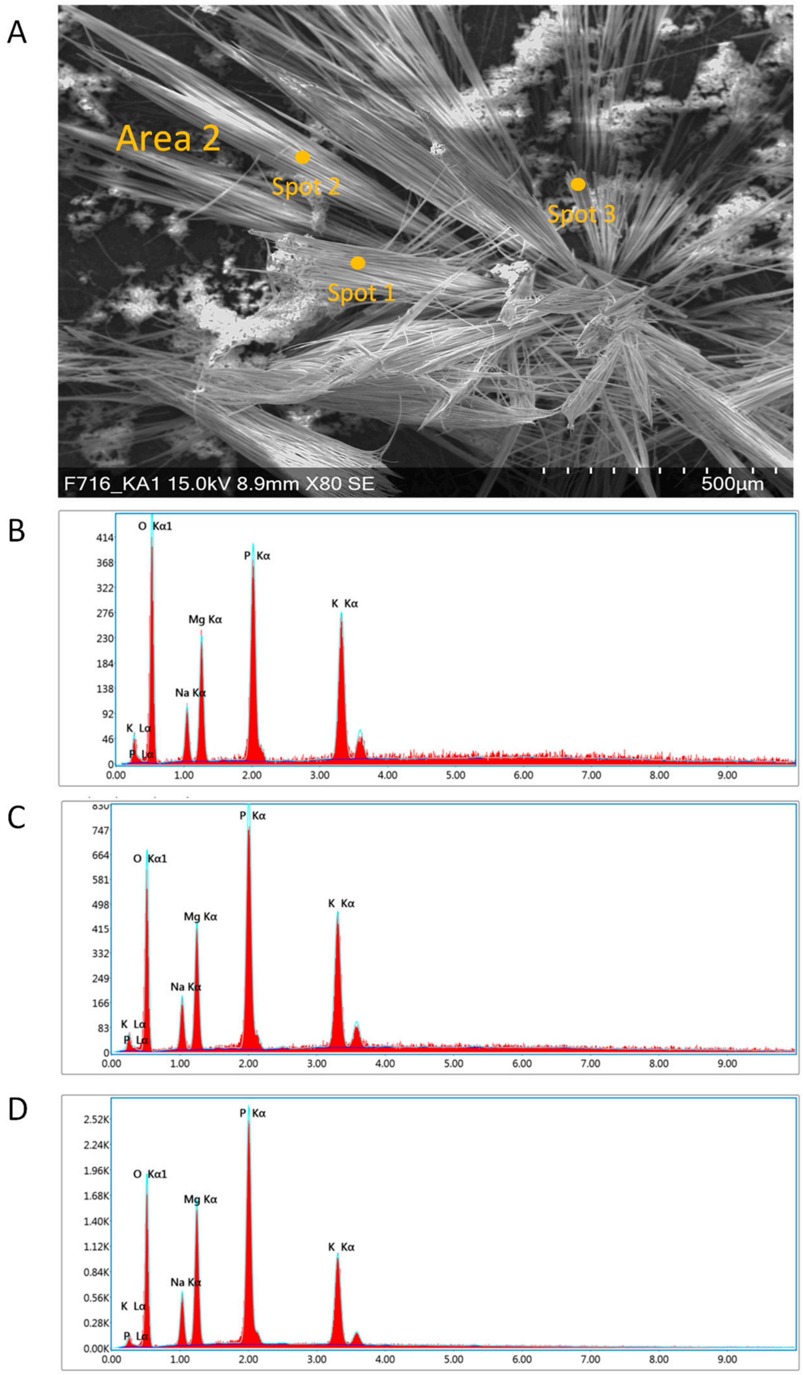

Figure S2. Element measurements at different spots of the electrode used during electromethanogenesis with *M. maripaludis* KA1. **(A)** SEM picture of electrode with deposits and distribution of the spots in area 2. **(B)** EDX analysis of spot 1, **(C)** EDX analysis of spot 2 and **(D)** EDX analysis of spot 3. The x-axis shows the energy in keV and the y-axis shows the total counts.

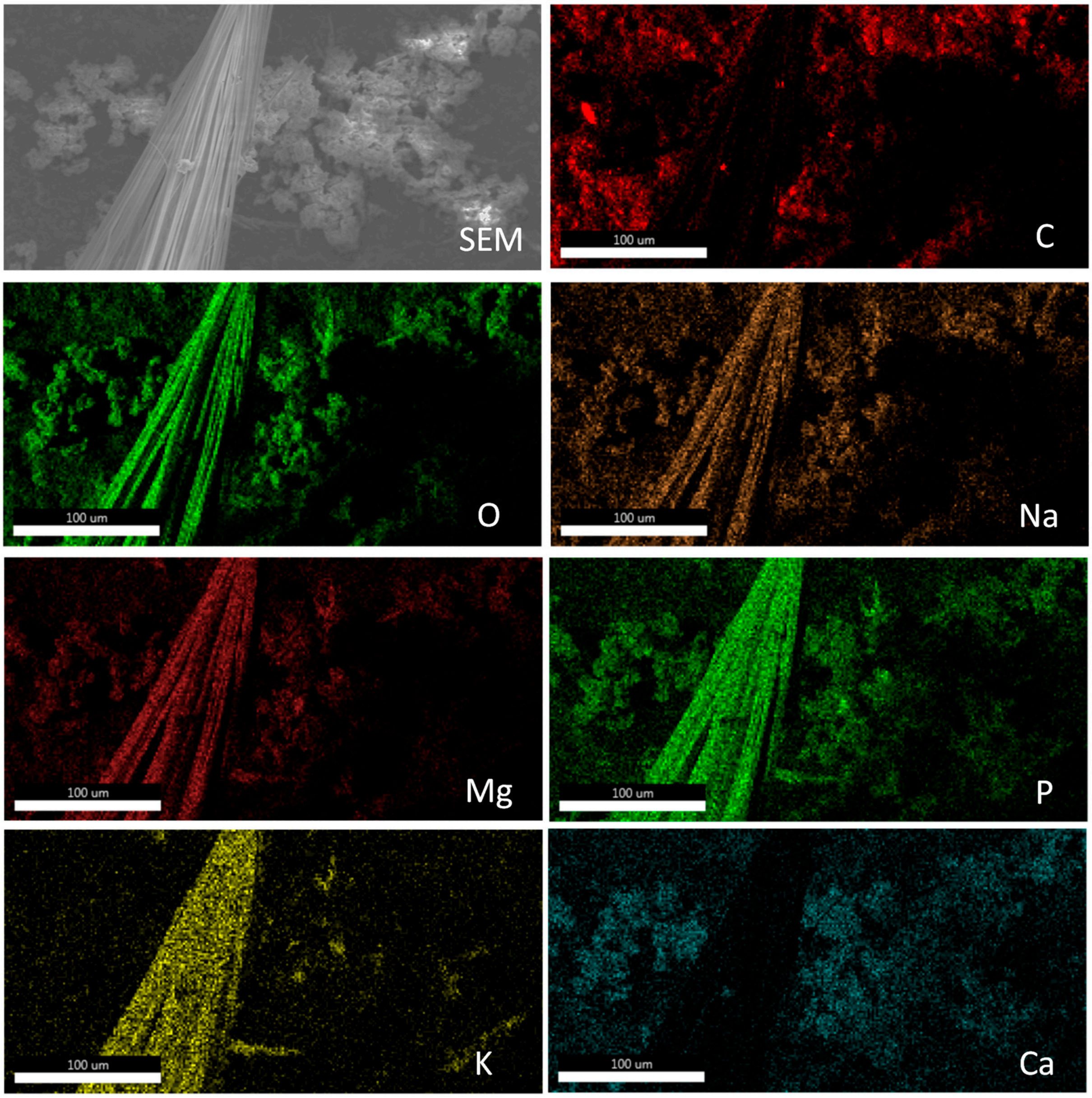

Figure S3. EDX mapping of an area with deposits of the electrode used during electromethanogenesis with *M. maripaludis* KA1. Different elements are shown in different colors.

Table S1. Additional genes in genome of *M. maripaludis* KA1 in comparison to strain S2.

**Locus tag KA1**	**Gene/protein**	
MMKA1_RS00465	4Fe-4S binding protein	Electron transport
MMKA1_RS00470	4Fe-4S binding protein	Electron transport
MMKA1_RS00475	4Fe-4S binding protein	Electron transport
MMKA1_RS00480	transcriptional regulator	
MMKA1_RS01060	hypothetical protein	
MMKA1_RS01065	hypothetical protein	
MMKA1_RS01290	hypothetical protein	
MMKA1_RS01295	hypothetical protein	
MMKA1_RS01300	DUF2341 domain-containing protein	
MMKA1_RS01305	hypothetical protein	
MMKA1_RS01310	DUF2341 domain-containing protein	
MMKA1_RS01700	hypothetical protein	
MMKA1_RS01875	UDP-glucose/GDP-mannose dehydrogenase family protein	
MMKA1_RS01920	TIGR04279 domain-containing protein	
MMKA1_RS01925	TIGR04279 domain-containing protein	
MMKA1_RS01930	hypothetical protein	
MMKA1_RS01935	polysaccharide biosynthesis protein	
MMKA1_RS01940	LegC family aminotransferase	
MMKA1_RS01945	Acetyltransferase	
MMKA1_RS01950	N-acetylneuraminate synthase	
MMKA1_RS01955	UDP-N-acetylglucosamine 2-epimerase (hydrolyzing)	
MMKA1_RS01960	acylneuraminate cytidylyltransferase family protein	
MMKA1_RS01965	Acyltransferase	
MMKA1_RS01970	Flippase	
MMKA1_RS01975	DUF1616 domain-containing protein	
MMKA1_RS01980	hypothetical protein	
MMKA1_RS01985	hypothetical protein	
MMKA1_RS01990	hypothetical protein	
MMKA1_RS01995	Glycosyltransferase	
MMKA1_RS02000	glycosyltransferase family 9 protein	
MMKA1_RS02005	ADP-glyceromanno-heptose 6-epimerase	
MMKA1_RS02010	HAD family hydrolases	
MMKA1_RS02015	bifunctional heptose 7-phosphate kinase/heptose 1-phosphate adenyltransferase	
MMKA1_RS02020	D-glycero-beta-D-manno-heptose 1-phosphate adenylyltransferase	
MMKA1_RS02025	D-sedoheptulose 7-phosphate isomerase	
MMKA1_RS02090	S-layer protein	
MMKA1_RS02305	tetratricopeptide repeat protein	
MMKA1_RS02310	hypothetical protein	
MMKA1_RS02345	hypothetical protein	
MMKA1_RS02500	hypothetical protein	
MMKA1_RS02505	hypothetical protein	
MMKA1_RS02510	hypothetical protein	
MMKA1_RS02515	hypothetical protein	
MMKA1_RS02520	hypothetical protein	

MMKA1_RS02525	tyrosine-type recombinase/integrase	
MMKA1_RS02530	hypothetical protein	
MMKA1_RS02535	hypothetical protein	
MMKA1_RS02540	hypothetical protein	
MMKA1_RS02545	hypothetical protein	
MMKA1_RS02550	hypothetical protein	
MMKA1_RS02555	helix-turn-helix domain-containing protein	
MMKA1_RS02560	hypothetical protein	
MMKA1_RS02565	tetratricopeptide repeat protein	
MMKA1_RS02570	hypothetical protein	
MMKA1_RS02575	hypothetical protein	
MMKA1_RS02580	hypothetical protein	
MMKA1_RS02585	recombinase family protein	
MMKA1_RS02590	hypothetical protein	
MMKA1_RS02595	hypothetical protein	
MMKA1_RS02600	ATP-binding protein	
MMKA1_RS02605	hypothetical protein	
MMKA1_RS02615	hypothetical protein	
MMKA1_RS02620	hypothetical protein	
MMKA1_RS02625	hypothetical protein	
MMKA1_RS02630	hypothetical protein	
MMKA1_RS02635	hypothetical protein	
MMKA1_RS02640	hypothetical protein	
MMKA1_RS02645	hypothetical protein	
MMKA1_RS02650	hypothetical protein	
MMKA1_RS02655	ABC transporter substrate-binding protein	
MMKA1_RS02660	ABC transporter ATP-binding protein	
MMKA1_RS02665	hypothetical protein	
MMKA1_RS02670	VWA domain-containing protein	
MMKA1_RS02675	AAA domain-containing protein	
MMKA1_RS02680	cobaltochelatase subunit CobN	
MMKA1_RS02685	hypothetical protein	
MMKA1_RS02690	hypothetical protein	
MMKA1_RS02695	hypothetical protein	
MMKA1_RS02700	PKD domain-containing protein	
MMKA1_RS02705	ABC transporter substrate-binding protein	
MMKA1_RS02710	iron ABC transporter permease	Diffusion of molecules
MMKA1_RS02715	iron ABC transporter permease	Diffusion of molecules
MMKA1_RS02720	TIM barrel protein	
MMKA1_RS02725	bifunctional adenosylcobinamide kinase/adenosylcobinamide-phosphate guanylyltransferase	
MMKA1_RS02730	MarR family transcriptional regulator	
MMKA1_RS02735	hypothetical protein	
MMKA1_RS02740	FRG domain-containing protein	
MMKA1_RS02745	hypothetical protein	
MMKA1_RS02750	hypothetical protein	
MMKA1_RS02820	hypothetical protein	
MMKA1_RS03130	hypothetical protein	
MMKA1_RS03330	class III signal peptide-containing protein	
MMKA1_RS03730	hypothetical protein	
MMKA1_RS04040	tRNA-Ser	

MMKA1_RS04045	site-specific integrase	
MMKA1_RS04050	hypothetical protein	
MMKA1_RS04055	hypothetical protein	
MMKA1_RS04060	site-specific integrase	
MMKA1_RS04065	DUF2540 domain-containing protein	
MMKA1_RS04070	hypothetical protein	
MMKA1_RS04075	nucleotidyltransferase family protein	
MMKA1_RS04080	nucleotidyltransferase family protein	
MMKA1_RS04085	Fic family protein	
MMKA1_RS04090	Nucleotidyltransferase	
MMKA1_RS04095	hypothetical protein	
MMKA1_RS04100	GIY-YIG nuclease family protein	
MMKA1_RS04105	winged helix DNA-binding protein	
MMKA1_RS04110	ATP-dependent DNA helicase	
MMKA1_RS04115	DEAD/DEAH box helicase	
MMKA1_RS04120	hypothetical protein	
MMKA1_RS04125	M48 family metallopeptidase	
MMKA1_RS04130	type I restriction endonuclease subunit R	
MMKA1_RS04135	hypothetical protein	
MMKA1_RS04140	hypothetical protein	
MMKA1_RS04145	type I restriction-modification system subunit M	
MMKA1_RS04150	restriction endonuclease subunit S	
MMKA1_RS04155	EVE domain-containing protein	
MMKA1_RS04160	hypothetical protein	
MMKA1_RS04165	hypothetical protein	
MMKA1_RS04170	DUF2975 domain-containing protein	
MMKA1_RS04175	hypothetical protein	
MMKA1_RS04180	DUF262 domain-containing protein	
MMKA1_RS04185	hypothetical protein	
MMKA1_RS04190	hypothetical protein	
MMKA1_RS04195	hypothetical protein	
MMKA1_RS04200	type II toxin-antitoxin system mRNA interferase toxin, RelE/StbE family	
MMKA1_RS04205	DUF3883 domain-containing protein	
MMKA1_RS04210	hypothetical protein	
MMKA1_RS04215	BREX system P-loop protein BrxC	
MMKA1_RS04220	DUF1819 family protein	
MMKA1_RS04225	CRISPR locus-related DNA-binding protein	
MMKA1_RS04230	ATP-dependent helicase	
MMKA1_RS04235	thermonuclease family protein	
MMKA1_RS04240	hypothetical protein	
MMKA1_RS04245	YjzC family protein	
MMKA1_RS04250	hypothetical protein	
MMKA1_RS04255	hypothetical protein	
MMKA1_RS04260	DUF4263 domain-containing protein	
MMKA1_RS04265	DEAD/DEAH box helicase	
MMKA1_RS04270	3'-5' exonuclease	
MMKA1_RS04275	hypothetical protein	
MMKA1_RS04280	recombinase family protein	
MMKA1_RS04285	hypothetical protein	
MMKA1_RS04290	DUF2683 family protein	
MMKA1_RS04295	hypothetical protein	
MMKA1_RS04300	AAA family ATPase	
MMKA1_RS04305	flavodoxin family protein	

MMKA1_RS04310	calcium/sodium antiporter	
MMKA1_RS04315	hypothetical protein	
MMKA1_RS04320	KamA family radical SAM protein	
MMKA1_RS04325	GNAT family N-acetyltransferase	
MMKA1_RS04330	site-specific integrase	
MMKA1_RS04335	hypothetical protein	
MMKA1_RS04340	ABC transporter permease	
MMKA1_RS04345	DUF4013 domain-containing protein	
MMKA1_RS04350	WYL domain-containing protein	
MMKA1_RS04355	hypothetical protein	
MMKA1_RS04360	trypsin-like peptidase domain-containing protein	
MMKA1_RS04365	hypothetical protein	
MMKA1_RS04370	hypothetical protein	
MMKA1_RS04435	flavodoxin family protein	
MMKA1_RS04500	radical SAM protein	
MMKA1_RS04505	hypothetical protein	
MMKA1_RS04510	hypothetical protein	
MMKA1_RS04515	RHS repeat-associated core domain-containing protein	
MMKA1_RS04520	hypothetical protein	
MMKA1_RS04525	RHS repeat-associated core domain-containing protein	
MMKA1_RS04530	hypothetical protein	
MMKA1_RS04535	hypothetical protein	
MMKA1_RS04540	hypothetical protein	
MMKA1_RS04545	hypothetical protein	
MMKA1_RS04550	hypothetical protein	
MMKA1_RS04555	hypothetical protein	
MMKA1_RS04560	hypothetical protein	
MMKA1_RS04565	RHS repeat-associated core domain-containing protein	
MMKA1_RS04570	hypothetical protein	
MMKA1_RS04575	hypothetical protein	
MMKA1_RS04580	hypothetical protein	
MMKA1_RS04630	formylmethanofuran dehydrogenase subunit B	
MMKA1_RS04730	N-6 DNA methylase	
MMKA1_RS04735	DUF2283 domain-containing protein	
MMKA1_RS04740	hypothetical protein	
MMKA1_RS04745	Integrase	
MMKA1_RS04875	hypothetical protein	
MMKA1_RS04880	hypothetical protein	
MMKA1_RS04885	hypothetical protein	
MMKA1_RS04920	S-layer protein	
MMKA1_RS05315	hypothetical protein	
MMKA1_RS05320	tyrosine-type recombinase/integrase	
MMKA1_RS05325	helix-turn-helix transcriptional regulator	
MMKA1_RS05330	hypothetical protein	
MMKA1_RS05335	hypothetical protein	
MMKA1_RS05340	hypothetical protein	
MMKA1_RS05345	CRISPR-associated endonuclease Cas6	
MMKA1_RS05350	CRISPR-associated helicase Cas3'	
MMKA1_RS05355	type I-B CRISPR-associated protein Cas5	
MMKA1_RS05360	type I-B CRISPR-associated protein Cas7/Csh2	
MMKA1_RS05365	TIGR02556 family CRISPR-associated protein	

MMKA1_RS05370	CRISPR-associated endonuclease Cas1	
MMKA1_RS05375	CRISPR-associated endonuclease Cas2	
MMKA1_RS05380	CRISPR-associated protein Cas4	
MMKA1_RS05450	formylmethanofuran dehydrogenase	
MMKA1_RS05455	FprA family A-type flavoprotein	
MMKA1_RS05460	YgiQ family radical SAM protein	
MMKA1_RS05465	phosphoenolpyruvate carboxylase	
MMKA1_RS05470	hydroxyethylthiazole kinase	
MMKA1_RS05475	thiamine phosphate synthase	
MMKA1_RS05480	hypothetical protein	
MMKA1_RS05485	formylmethanofuran dehydrogenase subunit A	
MMKA1_RS05490	formylmethanofuran dehydrogenase subunit C	
MMKA1_RS05495	4Fe-4S binding protein	Electron transport
MMKA1_RS05500	4Fe-4S dicluster domain-containing protein	Electron transport
MMKA1_RS05505	hypothetical protein	
MMKA1_RS05510	ATP-binding cassette domain-containing protein	
MMKA1_RS05515	hypothetical protein	
MMKA1_RS05520	formylmethanofuran dehydrogenase subunit B	
MMKA1_RS05525	formylmethanofuran dehydrogenase subunit B	
MMKA1_RS05530	hypothetical protein	
MMKA1_RS05535	CO dehydrogenase/acetyl-CoA synthase complex subunit epsilon	
MMKA1_RS05540	CO dehydrogenase/acetyl-CoA synthase complex subunit epsilon	
MMKA1_RS05545	threonylcarbamoyl-AMP synthase	
MMKA1_RS05550	4Fe-4S dicluster domain-containing protein	Electron transport
MMKA1_RS05555	hypothetical protein	
MMKA1_RS05560	2-oxoglutarate ferredoxin oxidoreductase subunitgamma	Ferredoxin-containing protein
MMKA1_RS05565	2-oxoacid:ferredoxin oxidoreductase subunit beta	Ferredoxin-containing protein
MMKA1_RS05570	2-oxoacid:acceptor oxidoreductase subunit alpha	Ferredoxin-containing protein
MMKA1_RS05575	ferredoxin family protein	Ferredoxin-containing protein
MMKA1_RS05580	4Fe-4S binding protein	Electron transport
MMKA1_RS05585	4Fe-4S binding protein	Electron transport
MMKA1_RS05590	5-(carboxyamino)imidazole ribonucleotide mutase	
MMKA1_RS05595	MTH1187 family thiamine-binding protein	
MMKA1_RS05600	carbonic anhydrase	
MMKA1_RS05605	phosphoribosylaminoimidazolesuccinocarboxamide synthase	
MMKA1_RS05610	chemotaxis protein	
MMKA1_RS05615	carbamoyl-phosphate synthase large subunit	
MMKA1_RS05620	glutamine-hydrolyzing carbamoyl-phosphate synthase small subunit	
MMKA1_RS05625	pyruvate synthase subunit beta	
MMKA1_RS05630	2-ketoisovalerate ferredoxin oxidoreductase subunit alpha (=porA)	Ferredoxin-containing protein
MMKA1_RS05635	4Fe-4S binding protein	Electron transport
MMKA1_RS05640	pyruvate ferredoxin oxidoreductase subunit gamma	
MMKA1_RS05645	Permease	
MMKA1_RS05650	4Fe-4S binding protein	Electron transport

MMKA1_RS05655	molybdate ABC transporter substrate-binding protein	
MMKA1_RS05825	hypothetical protein	
MMKA1_RS05830	hypothetical protein	
MMKA1_RS05835	hypothetical protein	
MMKA1_RS06040	N-6 DNA methylase	
MMKA1_RS06045	restriction endonuclease	
MMKA1_RS06145	hypothetical protein	
MMKA1_RS06150	hypothetical protein	
MMKA1_RS06155	endonuclease III	
MMKA1_RS06160	hypothetical protein	
MMKA1_RS06165	hypothetical protein	
MMKA1_RS06170	hypothetical protein	
MMKA1_RS06175	hypothetical protein	MIC island
MMKA1_RS06180	hypothetical protein	MIC island
MMKA1_RS06185	hydrogenase maturation protease	MIC island
MMKA1_RS06190	twin-arginine translocase subunit TatC	MIC island
MMKA1_RS06195	twin-arginine translocase TatA/TatE family subunit	MIC island
MMKA1_RS06200	carbonic anhydrase	MIC island
MMKA1_RS06205	PAS domain S-box protein	
MMKA1_RS06325	mannose-1-phosphate guanylyltransferase/ mannose-6-phosphate isomerase	
MMKA1_RS06330	glycosyltransferase family 4 protein	
MMKA1_RS06335	glycosyltransferase family 4 protein	
MMKA1_RS06340	glycosyltransferase family 2 protein	
MMKA1_RS06545	ABC transporter substrate-binding protein	
MMKA1_RS06550	ABC transporter permease	
MMKA1_RS06555	ABC transporter permease	
MMKA1_RS06560	ABC transporter ATP-binding protein	
MMKA1_RS06565	ATP-binding cassette domain-containing protein	
MMKA1_RS07110	cobalt-precorrin-7 (C(5))-methyltransferase	
MMKA1_RS07115	hypothetical protein	
MMKA1_RS07120	hypothetical protein	
MMKA1_RS07125	hypothetical protein	
MMKA1_RS07130	HAD family hydrolases	
MMKA1_RS07135	phosphoadenosine phosphosulfate reductase familyprotein	
MMKA1_RS07140	formate dehydrogenase accessory sulfurtransferase FdhD	
MMKA1_RS07145	HesA/MoeB/ThiF family protein	
MMKA1_RS07150	molybdenum cofactor biosynthesis protein MoaE	
MMKA1_RS07155	UPF0280 family protein	
MMKA1_RS07160	hypothetical protein	
MMKA1_RS07165	biotin synthase BioB	
MMKA1_RS07170	ATP-dependent sacrificial sulfur transferase LarE	
MMKA1_RS07175	O-phospho-L-seryl-tRNA:Cys-tRNA synthase	
MMKA1_RS07180	hypothetical protein	
MMKA1_RS07185	quinolinate synthase NadA	
MMKA1_RS07190	hypothetical protein	
MMKA1_RS07195	cobalt-precorrin-7 (C(5))-methyltransferase	
MMKA1_RS07200	hypothetical protein	
MMKA1_RS07205	hypothetical protein	
MMKA1_RS07210	hypothetical protein	
MMKA1_RS07215	HAD family hydrolases	

MMKA1_RS07220	phosphoadenosine phosphosulfate reductase familyprotein	
MMKA1_RS07225	formate dehydrogenase accessory sulfurtransferase FdhD	
MMKA1_RS07230	HesA/MoeB/ThiF family protein	
MMKA1_RS07235	molybdenum cofactor biosynthesis protein MoaE	
MMKA1_RS07240	UPF0280 family protein	
MMKA1_RS07245	hypothetical protein	
MMKA1_RS07250	biotin synthase BioB	
MMKA1_RS07255	ATP-dependent sacrificial sulfur transferase LarE	
MMKA1_RS07260	O-phospho-L-seryl-tRNA:Cys-tRNA synthase	
MMKA1_RS07265	hypothetical protein	
MMKA1_RS07270	quinolinate synthase NadA	
MMKA1_RS07275	hypothetical protein	
MMKA1_RS07280	cobalt-precorrin-7 (C(5))-methyltransferase	
MMKA1_RS07285	hypothetical protein	
MMKA1_RS07290	hypothetical protein	
MMKA1_RS07295	hypothetical protein	
MMKA1_RS07300	HAD family hydrolases	
MMKA1_RS07305	phosphoadenosine phosphosulfate reductase familyprotein	
MMKA1_RS07310	formate dehydrogenase accessory sulfurtransferase FdhD	
MMKA1_RS07315	HesA/MoeB/ThiF family protein	
MMKA1_RS07320	molybdenum cofactor biosynthesis protein MoaE	
MMKA1_RS07325	UPF0280 family protein	
MMKA1_RS07330	hypothetical protein	
MMKA1_RS07335	biotin synthase BioB	
MMKA1_RS07340	ATP-dependent sacrificial sulfur transferase LarE	
MMKA1_RS07345	O-phospho-L-seryl-tRNA:Cys-tRNA synthase	
MMKA1_RS07350	hypothetical protein	
MMKA1_RS07355	quinolinate synthase NadA	
MMKA1_RS07360	hypothetical protein	
MMKA1_RS08615	S-layer protein	
MMKA1_RS08705	amino acid ABC transporter substrate-binding protein	
MMKA1_RS08710	hypothetical protein	
MMKA1_RS08745	hypothetical protein	
MMKA1_RS08750	DUF2226 domain-containing protein	
MMKA1_RS08810	hypothetical protein	
MMKA1_RS09655	class III signal peptide-containing protein	
